# Transcriptional Regulation of Selenoprotein F by Heat Shock Factor 1 during Selenium Supplementation and Stress Response

**DOI:** 10.3390/cells8050479

**Published:** 2019-05-18

**Authors:** Bingyu Ren, Yanmei Huang, Chen Zou, Yingying Wu, Yuru Huang, Jiazuan Ni, Jing Tian

**Affiliations:** 1 Shenzhen Key Laboratory of Marine Biotechnology and Ecology, Department of Marine Biology, Shenzhen University, Shenzhen 518060, China; renbingyu89@foxmail.com (B.R.); jzni@szu.edu.cn (J.N.); 2 Changchun Institute of Applied Chemistry, Chinese Academy of Sciences, Changchun 130022, China; 3 College of Life Sciences and Oceanography, Shenzhen Key Laboratory of Microbial Genetic Engineering, Shenzhen University, Shenzhen 518060, China; 13534176785@163.com (Y.H.); chris_zou@163.com (C.Z.); 4 College of Life Sciences and Oceanography, Shenzhen Engineering Laboratory for Marine Algal Biotechnology, Shenzhen University, Shenzhen 518060, China; wing380323603@163.com (Y.W.); huangyuruby@163.com (Y.H.)

**Keywords:** Selenoprotein F, heat shock factor 1, transcription regulation, selenium supplementation, heat shock, ER stress

## Abstract

Changes of Selenoprotein F (SELENOF) protein levels have been reported during selenium supplementation, stressful, and pathological conditions. However, the mechanisms of how these external factors regulate SELENOF gene expression are largely unknown. In this study, HEK293T cells were chosen as an in vitro model. The 5′-flanking regions of SELENOF were analyzed for promoter features. Dual-Glo Luciferase assays were used to detect promoter activities. Putative binding sites of Heat Shock Factor 1 (HSF1) were predicted in silico and the associations were further proved by chromatin immunoprecipitation (ChIP) assay. Selenate and tunicamycin (Tm) treatment were used to induce SELENOF up-regulation. The fold changes in SELENOF expression and other relative proteins were analyzed by Q-PCR and western blot. Our results showed that selenate and Tm treatment up-regulated SELENOF at mRNA and protein levels. SELENOF 5′-flanking regions from −818 to −248 were identified as core positive regulatory element regions. Four putative HSF1 binding sites were predicted in regions from −1430 to −248, and six out of seven primers detected positive results in ChIP assay. HSF1 over-expression and heat shock activation increased the promoter activities, and mRNA and protein levels of SELENOF. Over-expression and knockdown of HSF1 showed transcriptional regulation effects on SELENOF during selenate and Tm treatment. In conclusion, HSF1 was discovered as one of the transcription factors that were associated with SELENOF 5′-flanking regions and mediated the up-regulation of SELENOF during selenate and Tm treatment. Our work has provided experimental data for the molecular mechanism of SELENOF gene regulation, as well as uncovered the involvement of HSF1 in selenotranscriptomic for the first time.

## 1. Introduction

As an essential trace element for mammals, selenium plays an important role in metabolism. Selenium deficiency is connected to several disorders, including cardiovascular, aging, and immune system diseases [1,2,3]. The biological function of selenium is achieved in the form of the 21st amino acid, selenocysteine (Sec) [4]. Proteins containing Sec are identified as selenoproteins. Selenium regulates the expression of selenoproteins, which are involved in a series of cellular defense responses, and thus protects cells against stressful stimuli like protein aggregates, heavy metal ions, thermal shock, and oxidative damage [5,6,7].

Selenoprotein F (SELENOF), the new name according to the recently published selenoprotein gene nomenclature, is also known by its former symbol the 15-kDa Selenoprotein (SEP15) [8]. It was first discovered by Gladyshev in 1998 [9]. The gene of SELENOF is located on chromosome 1p31, a locus where deletions or mutations are frequently discovered in cancer cases [10]. Current studies have revealed the connections between SELENOF polymorphisms and various risks of cancers, including colorectal cancer, lung cancer, breast cancer, and prostate cancer [11,12], yet the results from different populations can be conflicting and inconclusive. Both negative and positive regulating effects of SELENOF on tumor progression have been reported [13], indicating aberrant SELENOF expression may contribute to cancer pathologies.

As the gene product, SELENOF is an endoplasmic reticulum (ER) protein with N-terminal ER targeting sequences. Though no ER resident signal peptides were found in its sequence, SELENOF remains in the ER lumen by tight interaction with another ER protein UDP-glucose: glycoprotein glucosyltransferase (UGGT) [14]. Since it contains a thioredoxin-like domain in the C-terminal that exhibits redox activity, SELENOF may have thiol-disulfide oxidoreductase activity with the UGGT recognized misfolded proteins as its substrates [15]. A recently published study discovered that the secretions of disulfide-rich glycoproteins were affected by SELENOF deficiency, indicating a gatekeeper function of SELENOF in the redox quality control process of secreted glycoproteins [16]. The abnormal expression of SELENOF has been found in various diseases, like cancer and neurodegenerative disease. It is reported to be up-regulated in liver cancer cell lines [17] and down-regulated in the hippocampus and substantia nigra of Parkinson’s brain regions [18]. Since physiological importance of SELENOF has been revealed through understanding its function and relationship with certain diseases, it became more and more necessary for us to study the regulatory mechanisms behind.

Selenotranscriptomic studies have discovered several transcription factors that regulate the gene expression of selenoproteins, including NFκB, MTF1, HIF1, and the Sp family [19]. A few transcriptional studies solely dedicated to SELENOF have been reported. According to previous studies, the main effective ways to induce SELENOF expression regulation are through selenium compounds treatment and ER stress generation. SELENOF is highly dependent on selenium levels [20], and selenium supplementation significantly up-regulates mRNA level of SELENOF [21]. Also, as an ER protein that may be involved in protein folding control, SELENOF has been reported to be up-regulated during the tunicamycin (Tm) induced adaptive ER stress [22]. Thus, in the present study, the regulation effects of Heat Shock Factor 1 (HSF1) on SELENOF expression were investigated in selenate and Tm treated HEK293T cell models.

## 2. Materials and Methods

### 2.1. Promoter Analysis and Transcription Factor Binding Sites Prediction by Bioinformatics

The online Database of Transcriptional Start Sites (DBTSS) was used to analyze the 5′-flanking regions of Homo sapiens SELENOF (also known as SEP15) for promoter features [23,24]. Putative transcription factor binding sites for HSF1 was predicted online at JASPAR, an open-access database for eukaryotic transcription factor binding profiles [25,26]. The 5′-flanking regions (from −1430 to −248) were uploaded as promoter sequences and aligned with the JASPAR provided binding motif for over 70% similarity.

### 2.2. Plasmids Construction and Antibodies

A PCR-based method was used to generate all plasmid constructs used in this study. Sequence encoding full-length human HSF1 CDS (NM_005526), and a series of deletion derivatives from SELENOF gene 5′-flanking region sequences were amplified by PCR using human gDNA extracted from HEK293T as templates. HSF1 was inserted into the pcDNA3.1-Myc for overexpression in eukaryotic cells. The 5′-flanking region (from −2055 to +909) deletion derivatives were inserted into the pGL4.10-Luc2 luciferase vector for Dual-Glo Luciferase assay. All constructs were verified by sequencing.

Primary antibody for anti-HSF1 was purchased from abcam (ab52757), anti-myc-tag was purchased from Cell Signaling Technology (2276), anti-HA-tag was purchased from TransGen biotech (HT301-01), and anti- glyceraldehyde-3-phosphate dehydrogenase (GAPDH) was purchased from Abways (AB0036). Goat Anti-Rabbit IgG-HRP secondary antibodies were purchased from Abmart (M21002).

### 2.3. Cell Culture and Transfection

HEK293T and neuro-2a cell lines were used as cell models in our experiment. HEK293T cells were maintained in Dulbecco’s Modified Eagle’s Medium (DMEM, Hyclone) and supplied with 10% fetal bovine serum and 100 U/mL penicillin/streptomycin in a 5% CO_2_ 37 °C incubator. Neuro-2a cells were maintained in 50% DMEM, 45% OPTI-MEM medium supplied with 5% fetal bovine serum, and 100 U/mL penicillin/streptomycin in a 5% CO_2_ 37 °C incubator. Lipofectamine 2000 reagents were used for transfection with plasmid constructs and siRNAs, according to the manufacturer’s instructions. Specific siRNA to knock-down HSF1 (sc-35611) and its control siRNA (sc-36869) were purchased from Santa Cruz.

### 2.4. Cell Treatment

Heat shock stress was generated by maintaining the cells in a 43 °C-water bath for 1 h then recovered in the 37 °C incubator for 4 h. Selenium induced up-regulation of SELENOF was achieved by 1 μM sodium selenate treatment (Na_2_SeO_4_, Sigma S8295), with the time gradient from 0 to 72 h. The initial selenium concentration in cell culture medium was measured by CAS Test Technical Services Company (Guangzhou, China) using ICP-MS. The result showed that the selenium in cell culture medium was lower than the detection limit 0.1 ppm (mg/kg) (Supplementary Figure S1). Adaptive ER stress was induced by 5 μg/mL Tm treatment (Sigma T7765) with the time gradient from 0 to 24 h. Actinomycin D (Sigma 129935) was applied to the selenate or Tm pre-treated cells at a concentration of 1μg/mL for observing the mRNA degradation of SELENOF.

### 2.5. Dual-Glo Luciferase Assay

Cells cultured in 24-well plates were co-transfected with 380 ng pGL4.10 deletion derivatives (or 190 ng pGL4.10 deletion derivatives and 190 ng pcDNA3.1-HSF1) and 20 ng pRL-TK vectors per well. Selenate or Tm was added to the culture media simultaneously with the transfection when required. Luciferase assays were carried out 24 h after transfection using the Dual-Glo Luciferase Assay System from Promega according to the manufacturer’s instructions. The luminescence readings of Firefly and Renilla luciferase were measured by Luminoskan Ascent from Thermo Scientific.

### 2.6. Quantitative Real-Time PCR (Q-PCR)

Q-PCR was employed to confirm the gene expression of the identified proteins. A kit was used to extract total RNA from cells, according to the manufacturer’s instructions (Fastagen, RNAfast200). RNA templates were reverse transcribed to cDNA using a reverse transcription kit with gDNA eraser (Takara, RR047A). Q-PCR reactions were performed on the QuantStudio real-time PCR (ABI) with SYBR^®^ Green real-time PCR master mix (Toyobo, QPK-201). The following thermal cycling conditions were used: 5 min at 95 °C, followed by 40 cycles of 95 °C for 15 s and 60 °C for 1 min. The 2^−ΔΔCT^ method was applied to calculate the relative gene expression [27]. ACTB was chosen as an endogenous reference gene to normalize Q-PCR data. Primers for SELENOF were designed to amplify regions downstream of the UGA codon for Sec, so that transcripts containing the Sec codon were detected in our SELENOF mRNA results. Sequences of primer pairs for Q-PCR were listed in Supplementary Table S1.

### 2.7. Chromatin Immunoprecipitation (ChIP) Assay

HEK293T or Neuro-2a cells were incubated as above. Eighteen point five percent of formaldehyde was added into the cell culture medium to a final concentration of 1%, and then cells were treated at room temperature for 10 min to crosslink protein to DNA. ChIP assays were performed using an EZ-ChIP kit from Merck Millipore according to the manufacturer’s instructions. Aliquots of cross-linked chromatin were immunoprecipitated with anti-RNA polymerase II (positive control, provided by the kit), anti-HSF1 and normal rabbit IgG (negative control) antibody, respectively. To confirm the same amounts of chromatins used in immunoprecipitation between groups, input chromatin was used. The eluted DNA was amplified by PCR. Sequences of primer pairs for ChIP assay are listed in Supplementary Table S2.

### 2.8. Western Blotting

Whole cell extracts were prepared by lysing in 1 mL of lysis buffer (150 mM NaCl, 20 mM Tris, 1 mM EDTA, 1% Triton X-100, with protease inhibitors cocktail) on ice for 30 min and then ultrasonication. The lysates were spun at 20,000× *g* for 15 min at 4 °C and the supernatants were collected. A bicinchoninic acid (BCA) protein assay kit (Thermo, 23227) was used to measure the protein concentration for SDS-PAGE. Equal amounts of total proteins were loaded to the SDS-PAGE. Proteins were then transferred to a 0.22μm PVDF membrane (Merck Millipore) at 100 V for 90 min. Blots were blocked in Tris-buffered saline containing 0.1% tween (TBST) with 5% non-fat milk for 2 h, followed by incubating with the diluted primary antibodies at appropriate concentrations at 4 °C overnight. Blots were then washed for 15 min in TBST three times and subsequently incubated with the horseradish peroxidase-conjugated secondary antibodies at the appropriate concentration for 2 h at room temperature.

### 2.9. Data Analysis

The integrated densities of the blots were measured by Image J. Relative mRNA and protein levels quantification were presented as the ratio of target genes to the house-keeping genes. The relative luciferase activity quantification was presented as the ratio of firefly luciferase activity to the Renilla luciferase activity, according to the kit manufacturer’s instructions.

The Dual-Glo Luciferase assay and Q-PCR were performed three times independently with at least three technical replicates each time. The western blots were performed at least three times independently. Data are presented as mean ± SEM.

Student’s t-test were performed for comparisons between two groups and one-way ANOVA were performed for comparisons between multiple groups and time series treatment. * *p* < 0.05, ** *p* < 0.01 and *** *p* < 0.001 were considered to be statistically significant.

## 3. Results

### 3.1. Up-Regulation of SELENOF mRNA by Selenate and Tm Treatment

**Figure 1:**
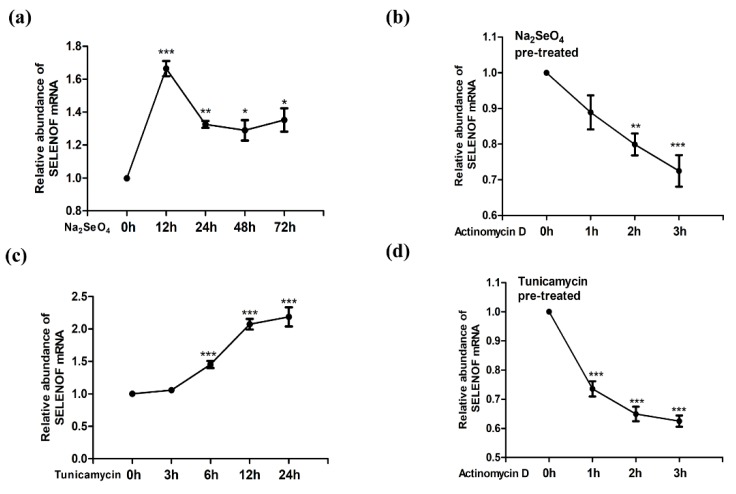
Selenoprotein F (SELENOF) mRNA up-regulation by selenate and tunicamycin (Tm). The transcription levels of SELENOF at several time intervals were quantified by Q-PCR in HEK293T cells (**a**) treated with selenate, (**b**) preconditioned with selenate then treated with Actinomycin D, (**c**) treated with Tm, and (**d**) preconditioned with Tm then treated with Actinomycin D. * *p* < 0.05, ** *p* < 0.01 and *** *p* < 0.001.

Several selenium compounds have been reported to be able to up-regulate SELENOF expression, including selenate, selenite, methylseleninic acid, and selenomethionine [28,29]. These selenium compounds, except selenate, are redox-reactive. ROS generation has been observed upon applying the redox-active selenium compounds [30], during which ER stress may be triggered [31]. Since SELENOF can also be up-regulated by ER stress, the non-redox-active selenate was used in our study for selenium supplementation. Also, selenate has been proofed with far less neurotoxicity than other forms of selenium [32]. According to a former report, up-regulation of SELENOF in cell lines can be induced by 50 nM~1 μM selenium compounds treatment for 72 h [33]. As shown in Figure 1a, 1 μM sodium selenate treatment induced a significant increase of SELENOF mRNAafter 12~72 h treatment in HEK293T cells. At the 12 h time point, the mRNA level reached a peak. The changes of SELENOF at transcriptional levels can be due to changes in transcription rate, changes in mRNA stability (degradation), or both. In order to observe the effect of selenium supplementation on SELENOF mRNA stability, a commonly used intercalating transcription inhibitor, Actinomycin D was applied to the selenate pre-treated (12 h interval) cells. Significant degradation of SELENOF mRNA was observed within 3 h (Figure 1b).

Our previous study found evidence of adaptive ER stress in 0.5~10 μg/mL Tm treatment for 24 h [34]. As shown in Figure 1c, 5 μg/mL Tm increased the mRNA level of SELENOF after 6 to 24 h of treatment. Unlike the change pattern of selenate treatment groups, a continuous increase of SELENOF mRNA within the 24-h treatment was observed in HEK293T cells. Actinomycin D caused notably SELENOF mRNA reduction to the Tm pre-treated (12 h interval) cells (Figure 1d). During the 1 h of Actinomycin D treatment, the absolute value of curve slope in Figure 1d (0.26) were greater than in Figure 1b (0.11), revealing that the SELENOF mRNA degradation rate in the Tm treated group was faster than in the selenate treated group.

### 3.2. Identification of the Core Positive Regulatory Element in 5′-Flanking Region of SELENOF

**Figure 2:**
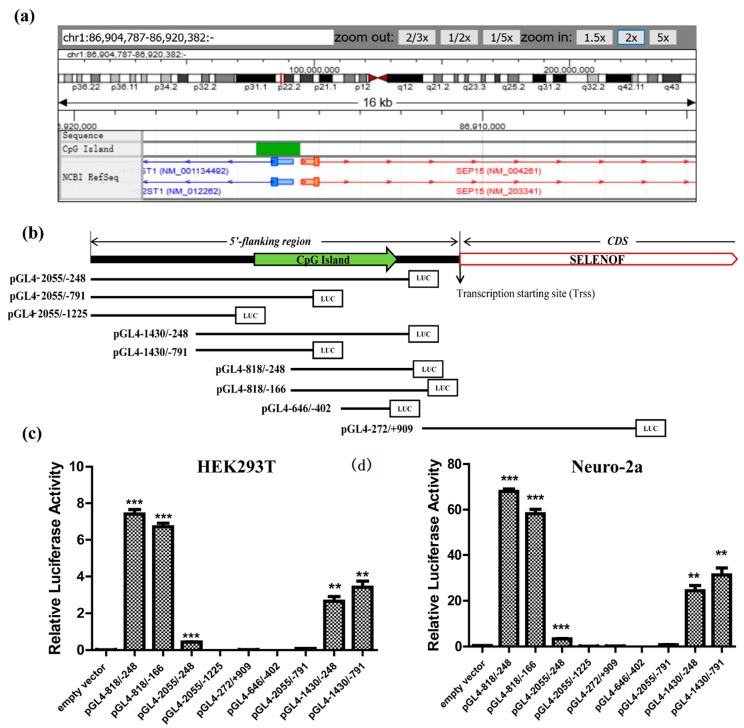
Identification of SELENOF regulatory elements. (**a**) Analysis of SELENOF (formerly known as SEP15) 5′-flanking regions in the Database of Transcriptional Start Sites (DBTSS). The SELENOF CDS was labeled with arrow and the CpG island was located up-stream of CDS. (**b**) Schematic locations of deletion derivatives designed for identification of SELENOF regulatory elements. Luciferase reporter activities of the deletion derivatives in HEK293T cells (**c**) and Neuro-2a cells (**d**). The empty vector pGL4.10 was used as the negative control. The data are representative of three independent experiments. * *p* < 0.05, ** *p* < 0.01 and *** *p* < 0.001.

The changes of SELENOF mRNA level during selenium and Tm treatment were consistent with the previous reports, yet the mechanism of SELENOF transcriptional regulation remained unknown. Transcription factors binding sites mainly exist in the upstream region of the transcription start site (TSS). To gain a preliminary understanding, we searched the DBTSS for the keyword SEP15 (the former name of SELENOF) in the Homo sapien species. As the results show in Figure 2a, a region with a high frequency of CpG sites—a typical CpG island, was found about 1kb upstream the TrSS, which was consistent with the in silico analysis from a former report [19]. The TSS-seq datasets of SELENOF in adult tissues and HEK293 cells are shown in the Supplementary Figure S2.

As CpG islands are commonly found in or near the promoters of mammalian genes [35], we selected the sequences from −2055 to +909 which covered the 5′-flanking regions of SELENOF gene containing the CpG islands. A series of deletion derivatives were designed for identification of the regulatory elements, and the schematic map of the fragments’ locations is shown in Figure 2b. Luciferase assays were carried out to study the promoter activities of the cloned DNA fragments in HEK293T. The deletion fragment from −818 to −248, which was the CpG sites enriched region, showed a significant promoter activity; while deletion fragments without the −818 to −248 sequences showed almost no promoter activity (Figure 2c). Similar activation profiles were further verified in Neuro-2a cell lines (Figure 2d). Thus, we identified the 5′-flanking region from −818 to −248 as a core positive regulatory element for SELENOF.

### 3.3. Increased Promotor Activities and Expression of SELENOF by HSF1 Over-Expression

SELENOF mRNA was up-regulated during selenate treatment and adaptive ER stress. Until now, transcription factors that have been identified to regulate the gene expression of selenoproteins are NFκB, MTF1, HIF1, the Sp family, and so on [19]. Also, plenty of transcription factors have been identified to take part in the ER stress response, involving ATF4, ATF6, Xbp1, HSF1, NFκB, AP1, SREBP, and CHOP [36,37,38]. In a previous study, we reported the regulation of SELENOF mRNA by NFκB [39]. Since HSF1 is reported to be able to compete with NFκB for DNA binding sites on the genome [40,41], we hypothesized that it may also be involved in the transcriptional regulation of SELENOF.

**Figure 3:**
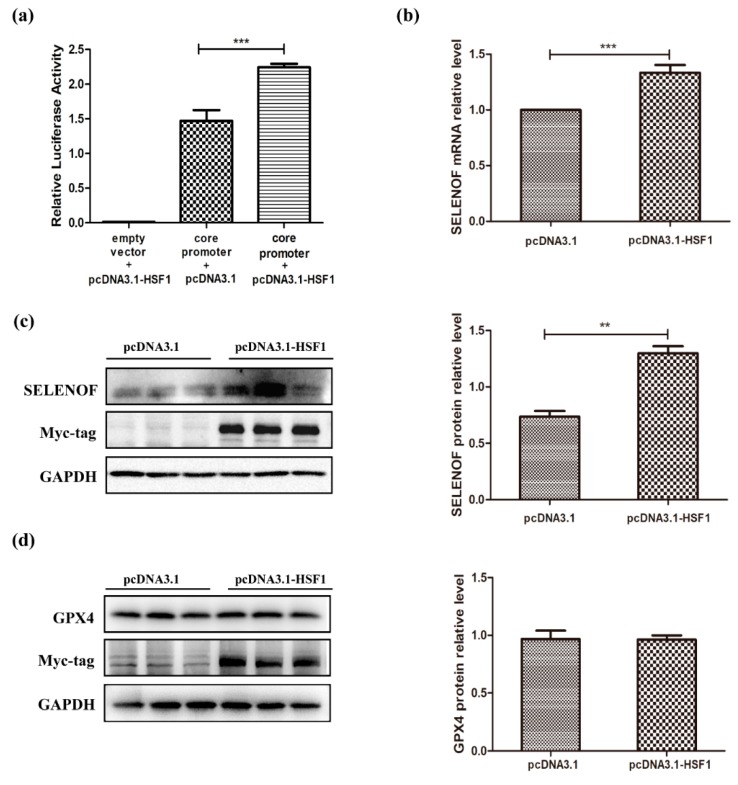
Heat Shock Factor 1 (HSF1) increased SELENOF transcriptional regulation. (**a**) Luciferase reporter activities of SELENOF core positive regulatory element, (**b**) transcriptional levels of SELENOF, protein levels of (**c**) SELENOF and (**d**) GPX4 in control and HSF1 over-expressed HEK293T cells. The empty vector pcDNA3.1 was used as the negative control to be compared with pcDNA3.1-HSF1. In Luciferase reporter assays, the empty vector pGL4.10 was used as the negative control, while the pGL4-818/-248 was used as core promoter of SELENOF. * *p* < 0.05, ** *p* < 0.01 and *** *p* < 0.001.

At first, the effects of HSF1 over-expression on SELENOF promoter activities and expression levels were studied. The pcDNA3.1 empty vector and pcDNA3.1-HSF1 plasmids were transfected into HEK293T cells separately. Over-expression of HSF1 not only significantly enhanced the promoter activities of SELENOF core positive regulatory element (Figure 3a), but also increased the mRNA (Figure 3b) and protein (Figure 3c) levels of SELENOF. Notably, HSF1 overexpression had no effect on the protein levels of another NFκB regulated selenoprotein GPX4 (Figure 3d), indicating the regulatory effect of HSF1 on SELENOF may be specific. These results validated our hypothesis preliminarily.

### 3.4. Recruitment of HSF1 to the SELENOF 5′-Flanking Regions

**Figure 4:**
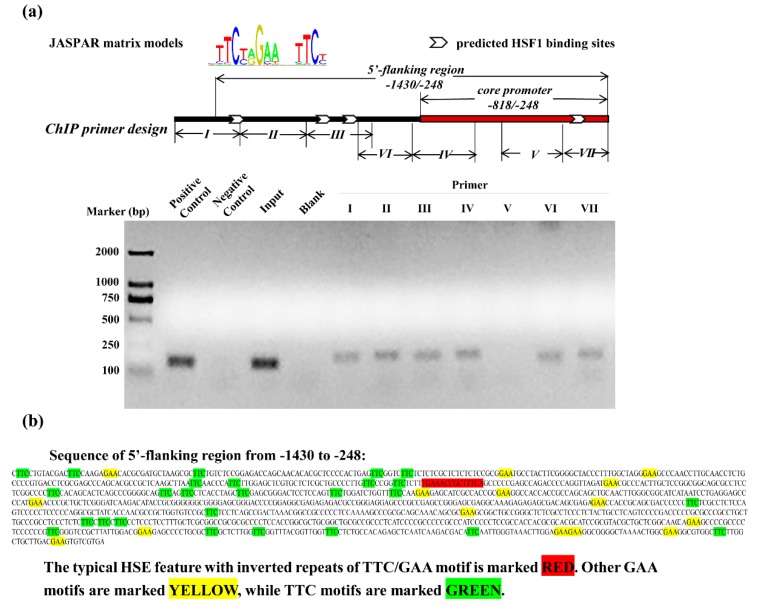
HSF1 interacted with the 5′-flanking regions of SELENOF. (**a**) PCR amplification results of chromatin immunoprecipitation (ChIP) primers after protein-chromatin cross-linkage and immunoprecipitation. Locations of JASPAR predicted HSF1 binding sites and the designed ChIP primers were presented in the schematic map. (**b**) Manually searched results of both typical and less conserved variant of HSF1 Sequence-binding Elements (HSEs) in the sequences of SELENOF 5′- flanking region from −1430 to −248. The data are representative of three independent experiments.

To determine whether HSF1 regulated the expression of SELENOF through associating with SELENOF 5′-flanking regulatory region, in silico analysis was performed in search of putative HSF1 transcription factor binding sites. The binding motif MA0486.1 from the online database JASPAR was applied to 5′-flanking region of SELENOF from −1430 to −248. Next, ChIP assays were used to verify the recruitment of HSF1 to SELENOF 5′-flanking regions. Relative locations of the predicted binding sites and ChIP primers were marked in the schematic map in Figure 4a. The PCR amplification of ChIP assays (from Line 6 to Line 12) showed positive results, except primer V (Line 10), which revealed the associations between HSF1 and various locations of SELENOF 5′-flanking region. These data suggested that there may be more HSF1 transcription factor binding sites than the JASPAR predicted results, as primer IV and VI also amplified obvious bands.

Furthermore, we searched the sequences from −1430 to −248 manually for HSF1 Sequence-binding Element (HSE). A typical HSE feature containing inverted repeats of the TTC/GAA motif with fixed spacing, 5′-tGAAaccgctTTCa-3′ was discovered from −1170 to −1156. Besides, TTC or GAA-rich sequence could be a less conserved variant of the HSE [42]. We also found numerous TTC or GAA motifs in the sequences from −1430 to −248 (Figure 4b), which may be the explanation for our ChIP result.

### 3.5. Activation of HSF1 by Heat Shock Treatment Up-Regulated the Promotor Activities and Expression of SELENOF

**Figure 5:**
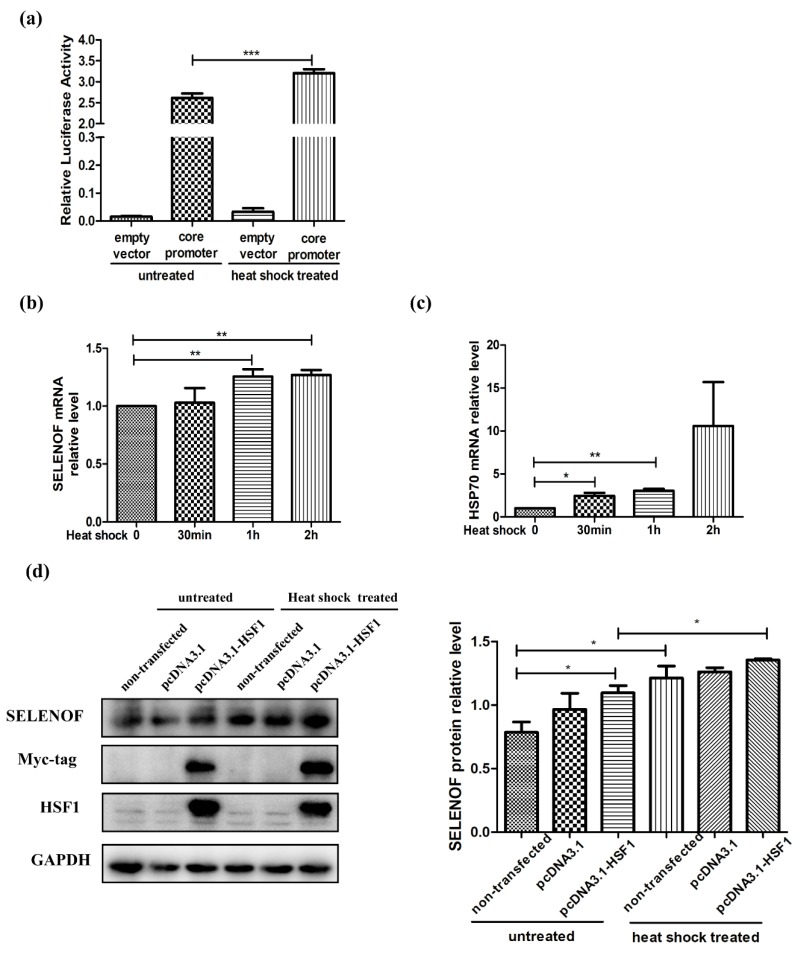
Heat shock treatment increased SELENOF transcriptional regulation. (**a**) Luciferase reporter activities of SELENOF core positive regulatory element in transfected HEK293T cells with/without heat shock treatment. Transcriptional levels of SELENOF (**b**) and heat shock protein (HSP)70 (**c**) at several time intervals in HEK293T cells under heat shock treatment. (**d**) Protein levels of SELENOF in control and HSF1 over-expressed HEK293T cells with/without heat shock treatment. The data are representative of three independent experiments. * *p* < 0.05, ** *p* < 0.01 and *** *p* < 0.001.

HSF1 was identified as a transcription factor that would be rapidly activated after stress, especially the temperature induced stress [43]. Hence, in the following experiments, we studied the effect of heat shock activation on the expression of SELENOF. The luciferase assay results were showed in Figure 5a. Heat shock treatment at 43 °C for 1 h enhanced the promoter activities of core positive regulatory element. Then Q-PCR were used to detect the mRNA levels at different time points after heat shock treatment. The heat shock protein (HSP)70 mRNA already increased after 30 min of heat shock (Figure 5c), while the SELENOF mRNA was up-regulated until 1 h treatment (Figure 5b). Western blot results in Figure 5d revealed that heat shock and HSF1 over-expression up-regulated the protein levels of SELENOF separately. Over-expression of HSF1 combined with heat shock treatment further slightly increased the SELENOF protein.

### 3.6. Endogenous Protein Changes of HSF1 and SELENOF During Selenate and Tm Treatment

**Figure 6:**
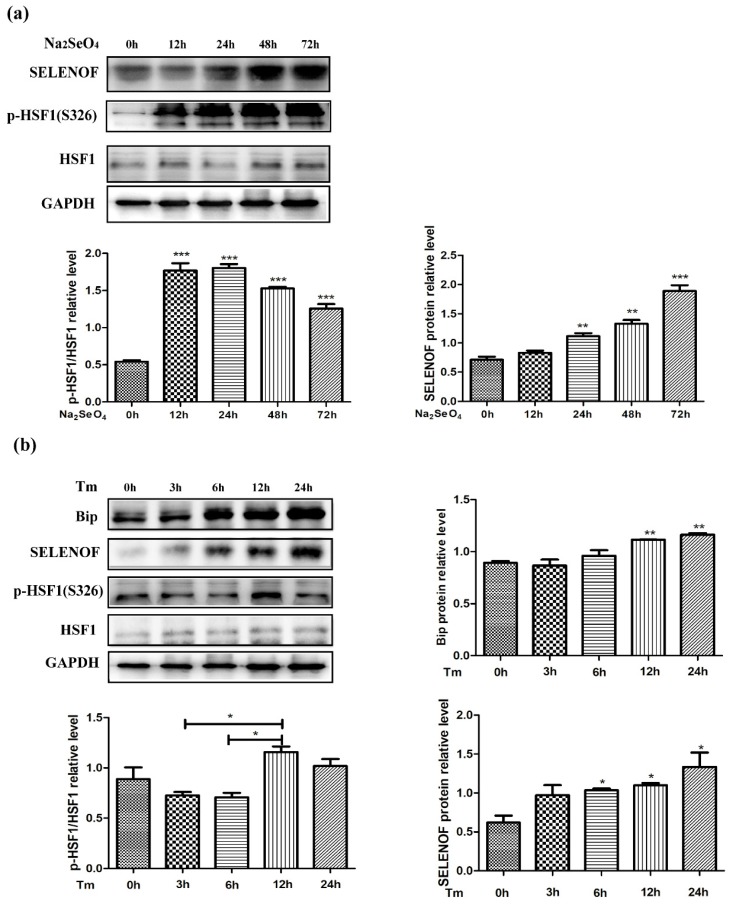
The effect of selenate and Tm treatment on the endogenous HSF1 activation and SELENOF protein levels. (**a**) Protein levels of SELENOF, p-HSF1 (S326), and HSF1 at several time intervals in HEK293T cells treated with selenate. (**b**) Protein levels of Binding immunoglobulin protein (BIP), SELENOF, p-HSF1 (S326), and HSF1 at several time intervals in HEK293T cells treated with Tm. BIP bands were used as protein markers for adaptive endoplasmic reticulum (ER) stress. The data are representative of three independent experiments. * *p* < 0.05, ** *p* < 0.01 and *** *p* < 0.001.

Unlike the change pattern of transcriptional levels in Figure 1a, the endogenous protein levels of SELENOF showed a continuous increase from 0 to 72 h in the selenate treated HEK293T cells (Figure 6a). Selenate showed minimal effect on the protein levels of total HSF1, but the phosphorylated HSF1 (p-HSF1 S326) significantly increased after 12 h selenate treatment, indicating the transcriptional activity of HSF1 (phosphorylated HSF1 S326/ total HSF1) was activated. Consistent with the Q-PCR results in Figure 1b, the protein levels of SELENOF were up-regulated after 6 h of Tm inducement in HEK293T cells (Figure 6b). The change pattern of SELENOF was similar to an ER chaperone Binding immunoglobulin protein (BIP, also called 78-kDa glucose-regulated protein GRP78), which was generally recognized as an adaptive ER stress marker. No obvious change of endogenous HSF1 expression was detected after the Tm treatment, while the ratio of phosphorylated HSF1 S326/ total HSF1 also significantly increased after 12 h of Tm treatment. In addition, we examined the endogenous mRNA and protein levels in Tm treated Neuro-2a cell lines. As shown in Supplementary Figure S3a,b, unlike the change pattern of BIP, SELENOF mRNA and protein levels first increased at 6 h and then decreased from 6 h to 24 h, which was quite different from the results that we obtained in HEK293T cells.

### 3.7. HSF1 Mediated Up-Regulation of SELENOF During Selenate and Tm Treatment

HSF1 is widely regarded as a transcription factor that mediates the expression of HSPs and other chaperones to facilitate protein folding and suppress protein aggregation. As an ER selenoprotein that was potentially useful during protein folding quality control, we speculated that SELENOF expression may be regulated by HSF1 during selenate treatment and adaptive ER stress.

**Figure 7:**
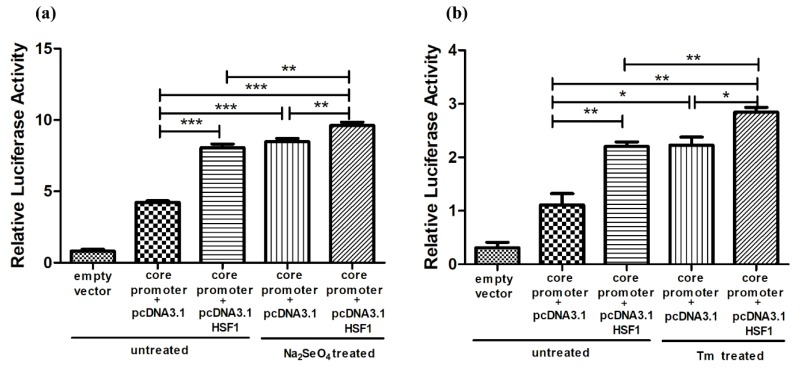
HSF1 further increased SELENOF transcriptional regulation during selenate and Tm treatment. Luciferase reporter activities of SELENOF core positive regulatory element in control and HSF1 over-expressed HEK293T cells with/without (**a**) selenate and (**b**) Tm treatment. * *p* < 0.05, ** *p* < 0.01 and *** *p* < 0.001.

Luciferase assay in Figure 7a revealed that the promoter activities of core positive regulatory element were doubled by selenate treatment for 24 h (2nd vs. 3rd bar) and HSF1 over-expression (2nd vs. 4th bar) separately. Selenate treatment combined with HSF1 over-expression further increased the promoter activities slightly (5th bar). Similar activation profiles were obtained in Tm treatment for 24 h (Figure 7b).

**Figure 8:**
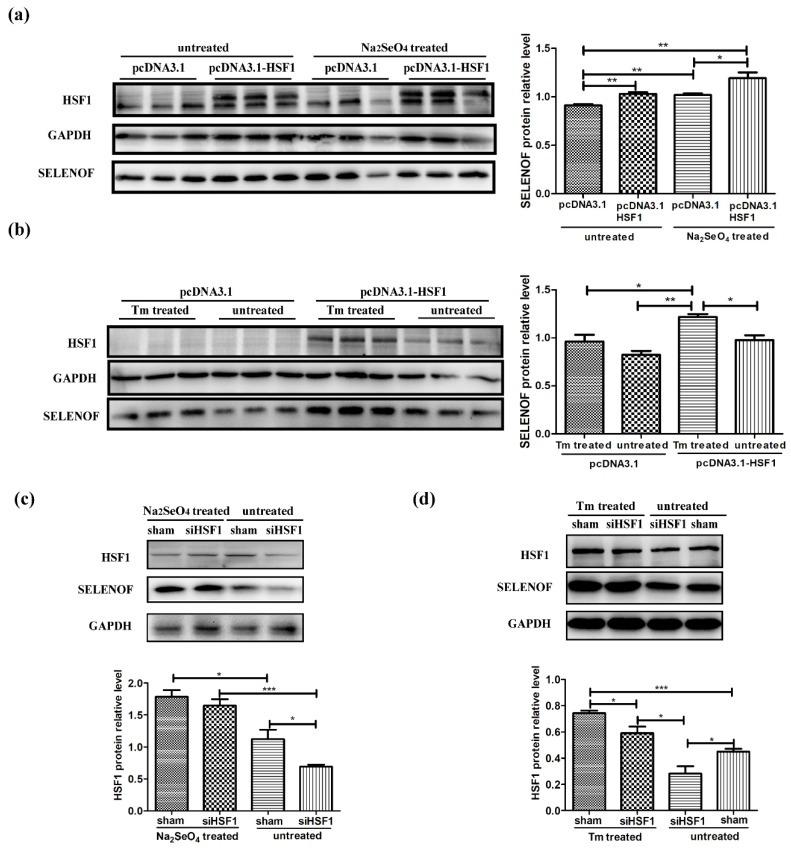
Involvement of HSF1 in the up-regulation of SELENOF during selenate and Tm treatment. Protein levels of SELENOF in (**a**) control and HSF1 over-expressed HEK293T cells with/without selenate treatment, (**b**) control and HSF1 over-expressed HEK293T cells with/without Tm treatment, (**c**) siRNA transfected HEK293T cells with/without selenate treatment, and (**d**) siRNA transfected HEK293T cells with/without Tm treatment. The data are representative of three independent experiments. The sham siRNAs were used as negative control to be compared with the siHSF1 siRNAs. * *p* < 0.05, ** *p* < 0.01 and *** *p* < 0.001.

Next, protein levels of SELENOF were detected and quantified by immunoblots in transfected HEK293T cells treated with selenate for 24 h (Figure 8a). SELENOF expression levels were up-regulated by over-expression of HSF1 (1st vs. 2nd bar) and selenate treatment (1st vs. 3rd bar), respectively. Moreover, HSF1 over-expression further increased the SELENOF protein levels in the selenate treated cells (3rd vs. 4th bar). Protein levels of HSF1 are shown in Supplementary Figure S3c. Over-expression resulted in obvious higher amounts of HSF1, while selenate treatment only slightly increased HSF1 on the basis of over-expression (with no statistical difference). Similar patterns were observed in HSF1 transiently transfected HEK293T cells treated with Tm for 24 h (Figure 8b and Supplementary Figure S3e). Finally, siRNAs were applied in HEK293T cells to knock down HSF1. HSF1 had been knocked down successfully in the untreated cells, while selenate and Tm treatment up-regulated the siRNA reduced HSF1 (Supplementary Figure S3d,f), as well as SELENOF (Figure 8c,d), which further indicated the involvement of HSF1 in up-regulation of SELENOF during selenate and Tm treatment.

## 4. Discussion

### 4.1. Specific Expression of SELENOF

As an ER protein, SELENOF is enriched in tissues with secretory functions, i.e., thyroid, liver, kidney, and reproductive organs, such as prostate and testis [44]. It is also abundantly expressed in brain regions like hippocampus and cerebellum [45]. We found that expression level of SELENOF exhibited different patterns induced by Tm treatment in HEK293T (Figure 1c and Figure 6a) and Neuro-2a cell lines (Supplementary Figure S3a,b). In a recently published study, cell-specific hierarchy of the selenoproteome has been confirmed in four different cell lines [46]. Thus, it can be assumed that the expression of SELENOF is tissue-specific or cell-type-specific.

In this study, HSF1 was identified as one of the transcription factors that have regulatory effect on SELENOF expression. During selenate and Tm treatment, the transcriptional activity of HSF1 was activated (Figure 6a,b), so there may be more activated HSF1 that were recruited to the SELENOF promoter. Notably, the transcriptional regulation of HSF1 may not be universal among all the selenoproteins. In three cell lines (LNCaP, HaCaT, and HepG2), another NFκB regulated selenoprotein GPX4 consistently ranks higher than SELENOF in the selenoproteome hierarchy in response to selenium supplementation [46]. However, over-expression of HSF1 only up-regulated SELENOF but had no effect on GPX4 (Figure 3c,d).

The specific expression of target gene is mediated by combination effects of both positive and negative regulatory elements [47]. Indeed, our luciferase assay results revealed not only the core positive regulatory element regions but also the existence of multiple negative regulatory elements to control SELENOF gene expression (Figure 2c,d). The region of −2055 to −1430 and −1430 to −818 may contain strong negative regulatory elements, as obvious repression effects were found among pGL4-2055/-248, pGL4-1430/-248, and pGL4-818/-248. The region of −248 to −166 may contain weak negative regulatory elements, as slight repression effects were found between pGL4-818/-166 and pGL4-818/-248. These identified elements may be involved in the cell- or tissue-specific expression of the SELENOF gene.

### 4.2. Regulation of SELENOF by Selenium Compounds at the Transcription and Translation Levels

The hierarchy of the selenoproteins in response to selenium has been identified in animal and cell models [46,48]. Generally, selenoproteins have been classified into two groups: those are resistant to selenium changes, also known as “house-keeping”; and those are sensitive to selenium changes, also known as “stress-regulated”. Interestingly, the mRNA of the typical “stress-regulated” selenoprotein GPX1 was found as natural substrates for nonsense mRNA degradation (NMD). In selenium deficiency cases, GPX1 mRNA may be degraded via NMD when the Sec-tRNAs were not sufficient enough to continue protein translation [49,50]. Similar to GPX1, SELENOF was recently ranked as a “stress-regulated” selenoprotein [46]. After new transcription inhibition by Actinomycin D, our results showed that the degradation of SELENOF mRNA was faster in the Tm treated group than in the selenate treated group (Figure 1b vs. Figure 1d), indicating that selenate may be able to stabilize SELENOF mRNA and prevent them from NMD degradation.

Notably, the up-regulation of SELENOF at protein levels were quite delayed compared to its mRNA levels. Significant increase of SELENOF protein levels was obtained from 24 h to 72 h after selenate treatment (Figure 6a), meanwhile no obvious change in SELENOF mRNA was detected during this period (Figure 1a). According to a former study, other selenium compounds, including selenite and methylseleninic acid, induced increase in protein levels of SELENOF with no mRNA change [33]. The up-regulation of SELENOF proteins were possibly the result of increasing translation of the still present SELENOF mRNA. The different change patterns of SELENOF at mRNA and protein levels in response to selenate treatment suggested that selenium compounds may affect the expression of SELENOF at both transcription and translation levels. Therefore, we speculated that during the “early stage” of selenate treatment, the sufficient Sec-tRNAs caused the burst of SELENOF mRNA increase by preventing them from degradation. Subsequently, as the Sec-tRNAs were continuously consumed by inserting into the newly synthesized SELENOF proteins, the mRNA levels would decrease, while its protein levels increased, until the dynamic equilibrium was achieved.

### 4.3. Potential Role of SELENOF in Selenium Supplementation and Stress Response

Antioxidative and stress-relieving effects of selenium compounds have been reported in several cellular stress conditions, including but not limited to heat shock response, oxidative stress, and ER stress [51,52,53]. It has been clarified that the antioxidative function of selenium, when supplied at rational levels corresponding to physiological optima, is through incorporating into selenoproteins with oxidoreductase functions [30]. Since SELENOF has been found to be sensitive to selenium changes and also potentially exhibit the thiol-disulfide oxidoreductase activity, it can be assumed that selenium may achieve its protective function by regulating SELENOF as one of the targets.

As classical molecular chaperones, the HSPs are activated by transcription factor HSF1 in temperature stress to prevent inappropriate folding and induce correct folding of proteins [54]. During the adaptive ER stress, a series of protective cellular actions, for example the Unfolded Protein Response, have been generated to restore the protein homeostasis in ER. The commonly accepted adaptive ER stress marker BIP is a chaperone protein that belongs to the HSP70 family [55]. Considering abnormal protein secretion or protein aggregation have been observed in SELENOF deficiency cases, together with our results that HSF1 up-regulated SELENOF during heat shock and adaptive ER stress, these indicated that SELENOF may play a protective role similar to the HSPs by handling the protein quality control.

## 5. Conclusions

As shown in the Graphical abstract, HSF1 was found to be associated with multiple sites of SELENOF 5′-flanking region. Over-expression or heat shock activation of HSF1 enhanced the promoter activities and expression of SELENOF. During selenate treatment and Tm induced adaptive ER stress, HSF1 was involved in the transcriptional up-regulation of SELENOF. Our results not only provided experimental data for SELENOF gene regulation but also uncovered the involvement of HSF1 in selenotranscriptomic for the first time.

## Supplementary Materials

The following are available online at https://www.mdpi.com/2073-4409/8/5/479/s1, Table S1: Sequences of primer pairs for Q-PCR (from 5′ to 3′), Table S2: Sequences of primer pairs for ChIP assay (from 5′ to 3′), Figure S1: ICP-MS detection of selenium in cell culture medium, Figure S2: TSS-seq datasets of SELENOF in adult tissues and HEK293 cells, Figure S3: The effect of Tm treatment on the transcription levels of *SELENOF* (**a**), and the endogenous protein levels of SELENOF, HSF1 and BIP (**b**) in neuro-2a cell lines. Quantification of HSF1 protein levels in HEK293T cells with/without 1 μM selenate treatment and HSF1 overexpression (**c**), 1 μM selenate treatment and HSF1 siRNA knockdown (**d**), 5 μg/mL Tm treatment and HSF1 overexpression (**e**), 5 μg/mL Tm treatment and HSF1 siRNA knockdown (**f**).
