# Supplementary material for: Transcriptional Regulation of Selenoprotein F by Heat Shock Factor 1 during Selenium Supplementation and Stress Response
Source: Cells. 2019 May 18;8(5):479. doi: 10.3390/cells8050479 (PMC6562903; doi:10.3390/cells8050479)
Supplement: Supplementary file 1 [file cells-08-00479-s001.pdf]

Supplementary data

Supplemental Table S1. Sequences of primer pairs for Q-PCR (from 5' to 3').

	Forward	Reverse
HPS70	AGAGCGGAGCCGACAGAG	CACCTTGCCGTGTTGGAA
SELENOF	AAGTCCAAGCTTTTGTTAGGAGTGATA	AAGCTTTAATACAGGGTCTGAACCA
ACTB	CCTGGCACCCAGCACAAT	GCTGATCCACATCTGCTGGAA

Supplementary data

Supplemental Table S2. Sequences of primer pairs for ChIP assay (from 5' to 3').

	Forward	Reverse
Input	GAAAAGGCGGGTCTCC GTGACG	GGTTCGCTCTGGGAAGCC TTGG
Primer I	CATTTCTGCGCACCTAT CCCTTC	GAGAGAGAAGACCGAAC TCAGTGG
Primer II	CCACTGAGTTCGGTCTT CTCTCTC	GAAAGCGGTTTCAAAGAG AACCGG
Primer III	CCGGTTCTCTTTGAAAC CGCTTTC	GGCGATGCTCTTCTTGGA AAACCAG
Primer IV	GCTGCTCGGGATCAAG ACATACC	GACACCAGCGGCGTTGGT GATAGC
Primer V	CTCTCGCCTCCCTCTAC TGCCTCA	GTTGCCGAGCAGCGTACG CGGATG
Primer IV	TTCAGTTCCTCACCTAG CTTCGAG	GGTATGTCTTGATCCCGAG CAGC
Primer VII	TTCGGGTCCGCTTATTG GACGGAAG	TCACGACACTTCGTCAAG CAGCCA

Supplementary data

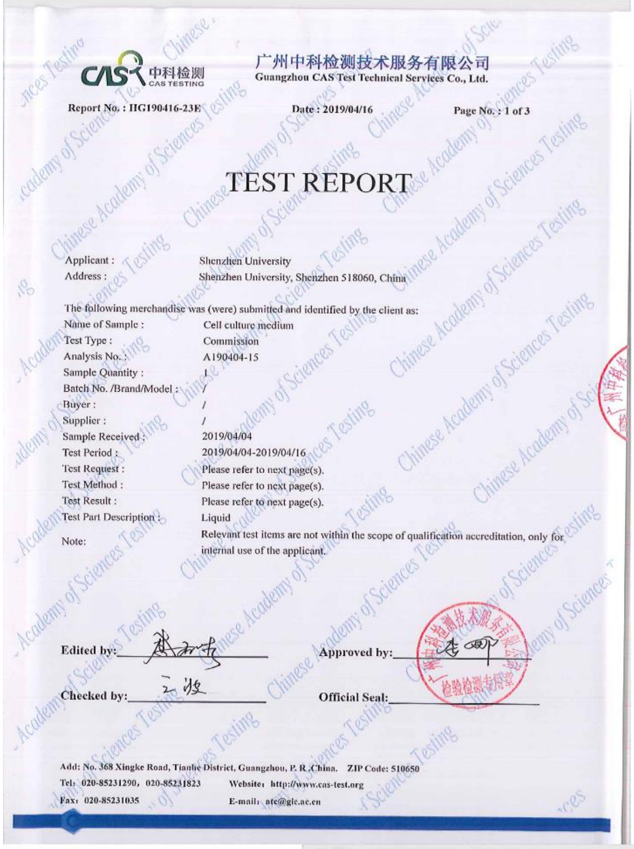

广州中科检测技术服务有限公司
Guangzhou CAS Test Technical Services Co., Ltd.

Report No. : HG190416-23E Date : 2019/04/16 Page No. : 1 of 3

TEST REPORT

Applicant :	Shenzhen University
Address :	Shenzhen University, Shenzhen 518060, China

The following merchandise was (were) submitted and identified by the client as:

Name of Sample :	Cell culture medium
Test Type :	Commission
Analysis No. :	A190404-15
Sample Quantity :	1
Batch No. /Brand/Model :	/
Buyer :	/
Supplier :	/
Sample Received :	2019/04/04
Test Period :	2019/04/04-2019/04/16
Test Request :	Please refer to next page(s).
Test Method :	Please refer to next page(s).
Test Result :	Please refer to next page(s).
Test Part Description :	Liquid
Note:	Relevant test items are not within the scope of qualification accreditation, only for internal use of the applicant.

Edited by:

Approved by:

Checked by:

Official Seal:

Add: No. 368 Xingke Road, Tianhe District, Guangzhou, P. R .China. ZIP Code: 510650
Tel: 020-85231290, 020-85231823 Website: http://www.cas-test.org
Fax: 020-85231035 E-mail: atc@gic.ac.cn

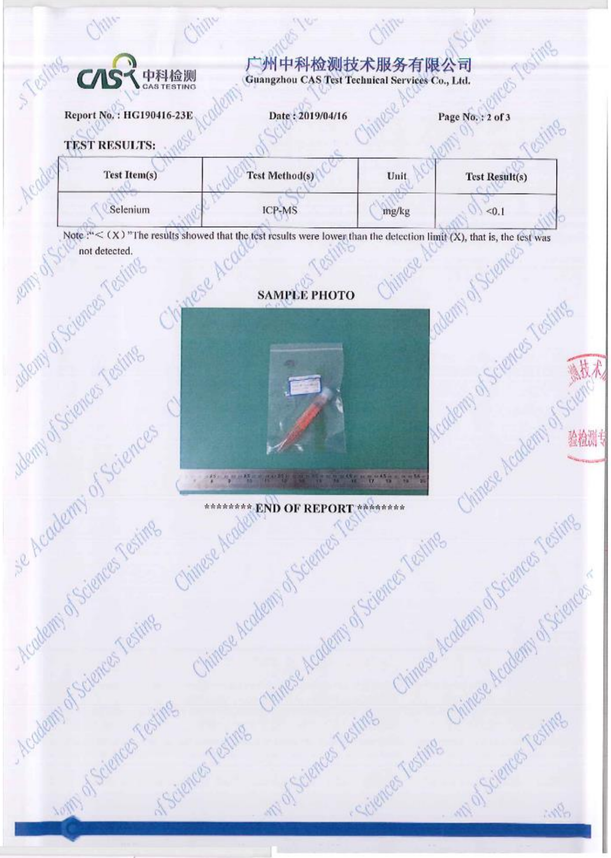

广州中科检测技术服务有限公司
Guangzhou CAS Test Technical Services Co., Ltd.

Report No. : HG190416-23E Date : 2019/04/16 Page No. : 2 of 3

TEST RESULTS:

Test Item(s)	Test Method(s)	Unit	Test Result(s)
Selenium	ICP-MS	mg/kg	<0.1

Note :"<（X）"The results showed that the test results were lower than the detection limit (X), that is, the test was not detected.

SAMPLE PHOTO

******** END OF REPORT ********

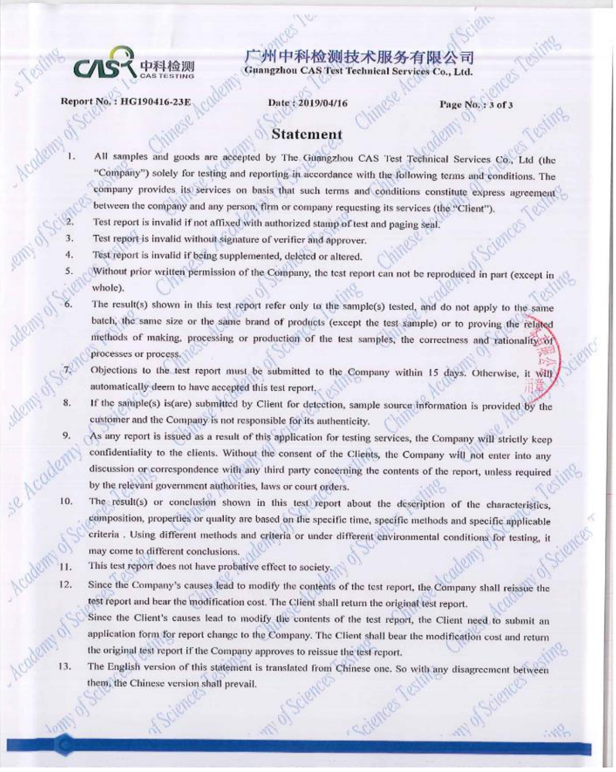

广州中科检测技术服务有限公司
Guangzhou CAS Test Technical Services Co., Ltd.

Report No. : HG190416-23E Date : 2019/04/16 Page No. : 3 of 3

Statement

1. All samples and goods are accepted by The Guangzhou CAS Test Technical Services Co., Ltd (the "Company") solely for testing and reporting in accordance with the following terms and conditions. The company provides its services on basis that such terms and conditions constitute express agreement between the company and any person, firm or company requesting its services (the "Client").
2. Test report is invalid if not affixed with authorized stamp of test and paging seal.
3. Test report is invalid without signature of verifier and approver.
4. Test report is invalid if being supplemented, deleted or altered.
5. Without prior written permission of the Company, the test report can not be reproduced in part (except in whole).
6. The result(s) shown in this test report refer only to the sample(s) tested, and do not apply to the same batch, the same size or the same brand of products (except the test sample) or to proving the related methods of making, processing or production of the test samples, the correctness and rationality of processes or process.
7. Objections to the test report must be submitted to the Company within 15 days. Otherwise, it will automatically deem to have accepted this test report.
8. If the sample(s) is(are) submitted by Client for detection, sample source information is provided by the customer and the Company is not responsible for its authenticity.
9. As any report is issued as a result of this application for testing services, the Company will strictly keep confidentiality to the clients. Without the consent of the Clients, the Company will not enter into any discussion or correspondence with any third party concerning the contents of the report, unless required by the relevant government authorities, laws or court orders.
10. The result(s) or conclusion shown in this test report about the description of the characteristics, composition, properties or quality are based on the specific time, specific methods and specific applicable criteria . Using different methods and criteria or under different environmental conditions for testing, it may come to different conclusions.
11. This test report does not have probative effect to society.
12. Since the Company's causes lead to modify the contents of the test report, the Company shall reissue the test report and bear the modification cost. The Client shall return the original test report.
    Since the Client's causes lead to modify the contents of the test report, the Client need to submit an application form for report change to the Company. The Client shall bear the modification cost and return the original test report if the Company approves to reissue the test report.
13. The English version of this statement is translated from Chinese one. So with any disagreement between them, the Chinese version shall prevail.

Supplemental Figure S1. ICP-MS test report for selenium in cell culture medium.

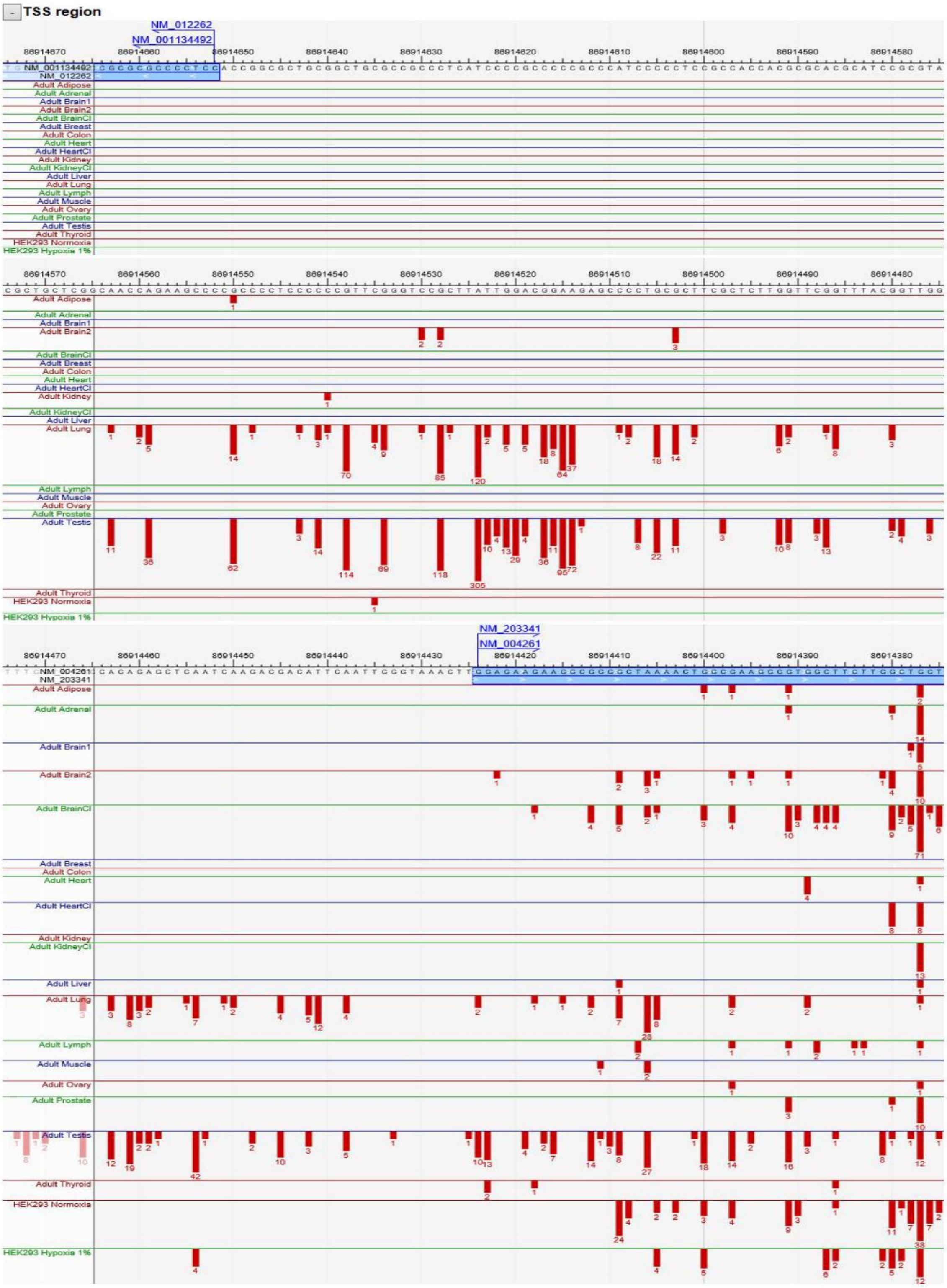

Supplemental Figure S2. TSS-seq datasets of *SELENOF* in adult tissues and HEK293 cells.

Supplementary data

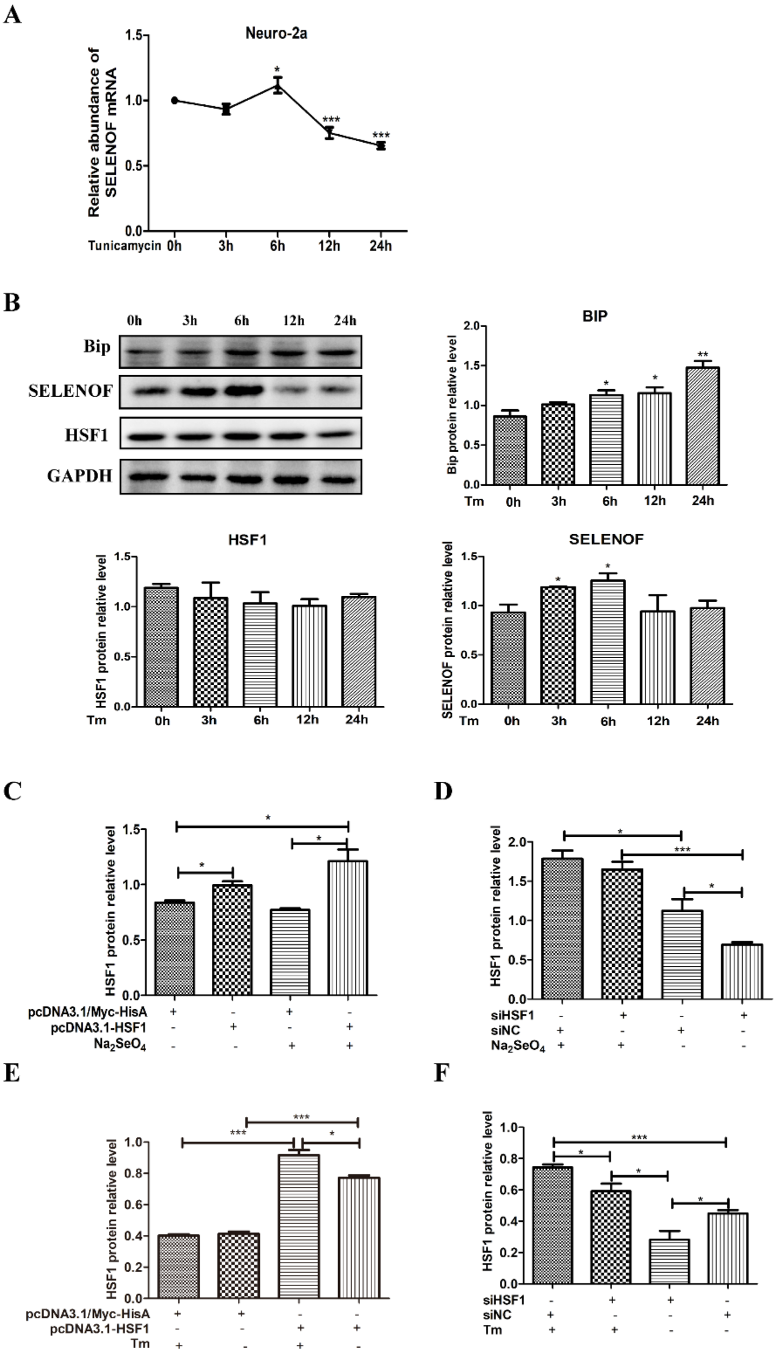

Supplemental Figure S3.

The effect of Tm treatment on the transcription levels of *SELENOF* (**a**), and the endogenous protein levels of SELENOF, HSF1 and BIP (**b**) in neuro-2a cell lines. Quantification of HSF1 protein levels in HEK293T cells with/without 1μM selenate treatment and HSF1 overexpression (**c**), 1μM selenate treatment and HSF1 siRNA knockdown (**d**), 5μg/mL Tm treatment and HSF1 overexpression (**e**), 5μg/mL Tm treatment and HSF1 siRNA knockdown (**f**).
